# HIV sexual risk behaviors and perception of risk among college students: implications for planning interventions

**DOI:** 10.1186/1471-2458-9-281

**Published:** 2009-08-04

**Authors:** Adedeji S Adefuye, Titilayo C Abiona, Joseph A Balogun, Mainza Lukobo-Durrell

**Affiliations:** 1 HIV/AIDS Research & Policy Institute, Chicago State University, 9501 S. King Drive, Chicago, IL 60628, USA; 2 Office of the Dean, Chicago State University, 9501 S. King Drive, Chicago, IL 60628, USA; 3 Jhpiego ‐ An affiliate of Johns Hopkins University, 8 Ngumbo Road, Long Acres Lusaka, Zambia

## Abstract

**Background:**

The college environment offers great opportunity for HIV high-risk behaviors, including unsafe sex and multiple partnerships. While the overall incidence of HIV infection has seen some decline in recent years, rates of HIV infection among young adults have not seen a proportionate decline. As in the general population, African American young adults have been disproportionately affected by the HIV/AIDS epidemic. This study examined the sexual risk behaviors and perception of HIV risk of students in a predominantly African American commuter urban university in the Midwest.

**Methods:**

Students enrolled in randomly selected general education courses completed a paper and pencil survey. Data were collected in Fall 2007, and univariate, bivariate, and multivariate analyses were conducted using SPSS for Windows v.16.

**Results:**

The sample included 390 students, the majority (83%) of whom were never married and 87% were sexually experienced. Among males reporting male partnerships those who used marijuana (OR = 17.5, p = 0.01) and those who used alcohol along with illegal drugs (OR = 8.8, p = 0.03) were significantly more likely to report multiple partnerships. Among females reporting male partnerships, those 30 years and older were significantly less likely (OR = 0.09, p = 0.03) to report having multiple male partners. There were significant differences in condom use last sex (p = 0.01) and consistent condom use (p = 0.002) among the different age groups. Older students were less likely to report condom use. Females age 30 years and older (OR = 3.74, p = 0.05) and respondents age 20‐29 years (OR = 2.41, p = 0.03) were more likely to report inconsistent condom use than those below 20 years. Marijuana use was correlated with inconsistent condom use (p = 0.02) and alcohol with not using condom last sex among females. Perception of HIV risk was generally poor with 54% of those age 30 years and older, 48.1% of 20‐29 year olds, and 57.9% of those below the age of 20 years perceived themselves as not having any chance of being infected with HIV. Predictors of moderate/good perception of HIV risk were drug and alcohol use, inconsistent condom use, and multiple partnerships.

**Conclusion:**

Students in the study sample engaged in various HIV risk behaviors but have a poor appreciation of their risk of HIV infection. While low rates of condom use was a problem among older students (30 years and older), multiple partnerships were more common among younger students, and marijuana and alcohol use were related to low condom use among females. Our findings support the need for targeted HIV prevention interventions on college campuses.

## Background

Recent statistics on the human immunodeficiency virus (HIV)/acquired immunodeficiency syndrome (AIDS) show a continuing racial disparity. While African Americans represent 12.3% of the U. S. population, they account for 50% of HIV/AIDS diagnoses and are more than 15 times more likely in their lifetime to be diagnosed with HIV compared to Whites [1]. The Centers for Disease Control and Prevention (CDC) in 2002 estimated that 1 in 50 African American men and 1 in 60 African American women are infected with HIV. The transmission rates among African Americans have not declined significantly in response to effective interventions compared with Whites [2]. In 2006, rates of AIDS cases were 47.6 per 100,000 for African Americans compared to 5.4 for Whites [3]. Although both African American men and women are disproportionately represented in HIV/AIDS diagnosis, the disparity is greatest for African American women who experience rates more than 20 times (40.4 per 100,000 vs. 1.9 per 100,000) that of White women [1,4].

Estimates show that 35% of new HIV infections among males and 32% of new HIV infections among females in the U.S. occur among individuals below the age of 29 years [5]. While the overall incidence of HIV infection has seen some decline in recent years, rates of HIV infection among young adults have not seen a proportionate decline [6]. At the end of 2004, an estimated 39,100 young adults ages 13‐24 in the U. S. received an AIDS diagnosis, representing 4% of the nation's total estimated cases [7]. As in the general population, African American young adults have been disproportionately affected by the HIV/AIDS epidemic.

A 2002 CDC report noted that the "epicenter of the HIV/AIDS epidemic is college students" [8]. According to the Census Bureau, in 2006, about 18 million students were enrolled in U.S. colleges [9]. While CDC statistics do not provide specific data for college students, a study by Gayle and others [10] estimated that 1 in 500 college students in the U.S. is infected with HIV. It is reasonable to speculate that the HIV/AIDS cases may be higher among African American college students due to the high prevalence of HIV infection among young African Americans in the general population [11]. In fact, a study conducted by Hightow and colleagues [12] found a trend for increasing rates of HIV infection for African American men attending colleges and universities.

African American women are most likely to be infected with HIV through high risk heterosexual contact [5]. They are often unaware of their male partners' risk factors for HIV infection, such as unprotected sex with multiple partners, bisexuality, or injection drug use [13,14]. Sexual contact is also the main risk factor for Black men. Male-to-male sexual contact was the primary risk factor for 48% of Black men with HIV/AIDS at the end of 2005, and high-risk heterosexual contact was the primary risk factor for 22% [15]. Substance users are more likely to engage in high-risk behaviors, such as unprotected sex, when they are under the influence of drugs or alcohol [16]. Sexually transmitted diseases (STDs), considered markers for HIV infection, are also more prevalent among African Americans. In 2005, African Americans were about 18 times as likely as Whites to have gonorrhea and about 5 times as likely to have syphilis [17]. Although African American students have increased risk of being infected with HIV as compared to White students, they do not manifest risk at the level that exists within their larger racial-ethnic community [18].

The college environment offers great opportunity for HIV high-risk behaviors, including unsafe sex [19]. College students are at risk because they tend to be sexually adventurous, often with multiple partners and do not consistently use condoms [20,21]. Jemmott and Jemmott [22] found that only 20% of sexually experienced, unmarried African American undergraduates at a commuter university always used condoms. African American women, who constitute the population with highest rate of new infections, are likely to be infected while in college [23].

Most studies on African Americans and HIV infection have focused on low-income, urban, or intravenous injection users [24-27]. Interventions developed for the African American general population may not be appropriate for African American college students. Studies conducted among African American college students [22,28,29], have focused mostly on condom use. In order to develop the body of knowledge necessary needed to develop interventions targeted to different ethnic/racial groups [20], it is necessary to study the overall HIV risk profile and perception of risk of African American college students, particularly their HIV risk behaviors in a variety of institutional settings. This approach is critical because of the plausible differences in risky behaviors between on-campus and off-campus (commuter) students. A 2001 study [30] found that a higher proportion of both male and female college students who stay off-campus report inconsistent condom use and two or more sexual partners compared with their on-campus colleagues. The study found that the two demographic variables that significantly predicted condom use among male and female students were age and student residence. Off-campus and older students had lower odds of consistent condom use.

The objective of this study is to determine the prevalence of HIV high risk behaviors and perception of HIV risk among students in a minority serving urban commuter university. As a sub-objective, we also explored the prevalence of HIV risky behaviors by sexual preference. In this article, we report high risk HIV risk behaviors and perception of risk of HIV infection among students in this minority institution, and discuss the implications of our findings for the planning of HIV prevention interventions for African American college students. This exploratory study provided the basis for the design and implementation of an HIV prevention intervention for students in this institution.

## Methods

### Sample and research design

Participants for this cross-sectional study were recruited from a minority serving commuter university in the Midwest. The sample included 390 students enrolled in 18 university general education courses. Because we were examining different risk behaviors, we used a prevalence rate of 50%, 95% CI and a 0.05 precision and arrived at minimal sample size of 384. Eighteen instructors from 34 classes that were randomly selected from the 133 general education courses published in the Fall 2007 course schedule responded to our email and telephone request and granted permission to conduct the survey in their classes. Following the instructors' consent, study information and data collection date was provided to students. Study participants ranged in age from 17‐64 years with a mean of 23.8 years. Thirty-two percent of the study participants were freshmen; 22% sophomores; 25% juniors; 17% seniors; and 3% undisclosed. The response rate was 93%.

### Research questionnaire

The investigators adapted survey instruments [31-34] that have been tested and used to conduct college based HIV/AIDS knowledge, attitudes, beliefs and behavior surveys. Authors selected relevant items from the following instruments: the HIV-Knowledge Questionnaire [30], the AIDS Attitude Scale [32], the National College Students Health Risk Behavior Survey [33], and the National Health Interview Survey of AIDS Knowledge and Attitudes [34].

In this manuscript, only the data relating to sexual risk behaviors and perception of HIV risk are presented. The development and testing of the psychometric properties of the research questionnaire is presented elsewhere.

### Measures

We defined "sexually experienced" as having previously or currently engage in any form of sex. The two HIV-risk sexual behaviors of multiple sexual partners and inconsistent or non-use of condoms were chosen from CDC's list of risk factors for HIV transmission [35]. Consistency of condom use was assessed by the question: "During the past 30 days, how often did you or your partner use a condom? Always was scored "0", any other answer was scored "1"(risky sex) To assess consistency of condom use among sexually experienced individuals who did not have sex within the last 30 days prior to the study, the answer from another question ‐ "The last time you had sexual intercourse, did you or your partner use a condom" ‐ was used. Self-perception of HIV risk was assessed by the question "what are the chances that you might catch HIV? Would you say there is no chance, a moderate chance or a good chance?"

### Procedure

Approval to carry out the study was obtained from the Institutional Review Board (IRB) at Chicago State University. On the scheduled data collection dates, a recruitment script approved by the IRB was read to the students followed by an informed consent script for those who volunteered to participate. The recruitment script explained the purpose, significance, benefits and potential risks of the study. The informed consent script stated that participation in the study was anonymous and voluntary and non-participation would have no social or academic consequences. Students who volunteered to participate were then asked to complete a paper and pencil survey after providing verbal consent. Upon completion, each student put the survey in a sealed envelope and dropped it in a box provided by the research assistant, and received a $10 bookstore voucher.

### Data analysis

The data were analyzed using the Statistical Package for Social Sciences (SPSS) version 16. Data was summarized using descriptive and inferential statistics. HIV risk behaviors and perception of the risk of HIV infection were compared between the different age groups using the Chi-square (χ^2^) tests. The association between several independent variables (age, gender, ethnicity, marital status, membership of fraternity, age at sexual debut, use of alcohol, marijuana, cocaine and other illegal drugs in the previous 30 days and in the context of sex) and the dependent variables (condom use during last sexual intercourse, frequency of condom during sexual intercourse in the last 30 days, number sexual partners in the last 3 months, and perceived risk of HIV infection) were ascertained using bivariate and multivariate logistic regression analysis. Data was stratified by gender and sexual preference when necessary. For the logistic regression, independent variables with *P *values of 0.25 [36] and above in the bivariate analysis were entered simultaneously into a multivariate model. Odds ratios (ORs) and 95% confidence intervals (CIs) were calculated.

## Results

### Demographic profile of the sample

Table 1 shows the demographic and other characteristics of the study sample. Of the 390 study participants, 30.3% were male, 69% were female, and 0.8% provided no response; 29.3% were below the age of 20 years, 46.7 were between 20 and 29 years, 12.8 were 30 years and older, and 11.3% did not indicate their age. The racial/ethnic profile of the sample as depicted in Table 1 mirrored the demographic characteristics of the institution (78.6% African American, 7.4% Hispanic/Latino, 9.1% White, 1.1% Asian/Pacific Islander, 0.5% Native American, and 3.7% "other.") This suggests that the sample is representative of the institution's student population.

**Table 1:** Demographic characteristics of the respondents (N = 390)

Categories	n	%
**Age**		
< 20 Years	114	29.3
20‐29 Years	182	46.7
30 Years and Above	50	12.8
No Response	44	11.3
		
**Gender**		
Female	269	69.0
Male	118	30.3
No response	3	0.8
		
**Race/Ethnicity**		
White	26	6.7
African American	313	80.3
Hispanic or Latino	29	7.4
Others	22	5.7
		
**Sexually experienced**		
Yes	339	86.9
No	51	13.1
		
**Marital status**		
Never been married	324	83.1
Married	26	6.7
Separated	7	1.8
Divorced	21	5.4
Widowed	11	2.8
No response	1	0.3
		
**Relationship preferences**		
Male Respondent with Male Partners	33	8.5
Male Respondents with Female Partners	57	14.6
Female Respondent with Male Partners	164	42.1
Female Respondents with Female Partners	38	9.7
No Response	98	25.1
		
**Member of a social fraternity or sorority**		
Yes	36	9.2
No	352	90.3
No response	1	0.3
		
**Enrollment status**		
Full-time	341	87.4
Part-time	49	12.6
		
**Current residence**		
University residence hall (dormitory)	69	17.7
Fraternity or sorority house	14	3.6
Other university housing	4	1.0
Off-campus house or apartment	165	42.3
Parent/guardian's home	125	32.1
Other	13	3.3

The majority of the sample were never married (83.1%), nor belong to any fraternity or sorority (90.3%). Eighty seven percent (87%) were enrolled full-time and less than 20% resided in the university dormitory.

### Initiation of sexual activity

The sexual behaviors of the respondents and factors associated with having multiple partners in the last 3 months are presented in Tables 2 and 3, respectively. About 87% of the sample reported being sexually experienced. Among the sexually experienced, 27.1% had their sexual début before the age of 14 years or younger, 57.8% between ages 15 and 18 years, 10.6% at age 19 years or older; 4.4% provided no responses. The breakdown by age is presented in Table 2.

**Table 2:** Sexual behaviors and perception of HIV risk of sexually experienced respondents by age (N = 339)

Categories	Age group			p-value
		
	0‐19 Years (n = 96)	20‐29 Years (n = 164)	30 Years and Above (n = 48)	
**Age at sexual debut**				
14 or less	33.0	27.2	14.3	
15‐18	56.0	60.1	76.2	0.20
19 and above	11.0	12.7	11.7	
				
**Frequency of sexual intercourse in last 30 days**				
0	39.1	32.2	27.1	
Once	8.2	12.8	6.3	
2‐3 times	17.3	9.4	16.7	0.15
4‐9 times	17.3	23.3	31.3	
10‐19 times	9.1	12.2	14.6	
20 or more times	9.1	10.0	4.2	
				
**Number of male sexual partners in the past 3 months**				
***Male students***				
1	83.3	78.9	100.0	0.67
2 or more	16.7	21.2	0	
***Female students***				
1	68.8	73.2	96.4	0.02
2 or more	31.3	26.8	3.6	
				
**Number of female sexual partners in the past 3 months**				
***Male students***				
1	63.6	52.0	71.4	0.60
2 or more	36.4	48.0	28.6	
***Female students***				
1	54.5	55.0	75.0	0.75
2 or more	45.5	45.0	25.0	
				
**Condom use during last sexual intercourse**				
Yes	51.5	39.0	25.0	0.01
No	48.5	61.0	75.0	
				
**Condom use during sexual intercourse in last 30 days**				
Never/Rarely	42.3	55.8	78.4	
Sometimes/Most of the time	21.8	24.0	8.1	0.002
Always	35.9	20.2	13.5	
				
**Alcohol and/or drug use during last sexual intercourse**				
Yes	14.6	13.8	4.9	0.26
No	85.4	86.2	95.1	
				
**Perceived chances of HIV infection**				
No Chance	57.9	48.1	54.0	
Moderate Chance	25.4	30.9	32.0	0.35
Good Chance	4.4	8.3	4.0	
Already Infected	0.9	0	0	
Don't Know/No Response	11.4	12.7	10.0	

### Frequency of sexual intercourse

Among the sexually experienced participants, 18.2% of those under the age of 20 years, 22.2% between 20 and 29 years, and 18.8% of those 30 years and over reported having sexual intercourse 10 or more times in the 30 days before the survey (Table 2).

### Number of sexual partners

Among the sexually experienced males who reported having male sexual partners in the past 3 months, 16.7% of those below the age of 20 years and 21.2% of those age 20 to 29 years reported having 2 or more sexual partners in the previous 3 months. No respondent 30 years and over reported multiple partners in the previous 3 months (Table 2). In this group, males who used marijuana (OR = 17.5, p = 0.01) and those who used alcohol along with illegal drugs (OR = 8.8, p = 0.03) were significantly more likely to have had more than one partner in the previous 3 months (Table 3).

**Table 3:** Factors associated with having multiple sexual partners in the previous 3 months

Variables	n/N*	(%)	Unadjusted Odds Ratio (95% CI)	P-Value
	**Females Reporting Male Partnerships**			
				
**Age (Years)**				
< 20	12/46	(28.3)	1	
20‐29	22/82	(26.8)	0.93 (0.42‐2.09)	0.86
30 & Above	1/28	(35.7)	0.09 (0.01‐0.76)	0.03
				
**Ethnicity**				
White	1/7	(14.3)	1	
African American	25/134	(18.7)	1.73 (0.20‐14.94)	0.62
Hispanic or Latino	3/12	(25.0)	4.29 (0.39 ‐ 47.63)	0.24
Others	0/10	(0.00)	0.00 (0.00 ‐ 0.00)	1.00
				
**Marital status**				
Never been married	26/135	(19.3)	1	
Married	0/11	(0.00)	0.00 (0.00‐0.00)	1.00
Separated/Divorced/Widow	3/17	(17.6)	0.70 (0.18 ‐ 2.45)	0.54
				
**5 or more drinks of alcohol in last 30 days**				
0 days	21/120	(17.5)	1	
1 or more days	8/37	(21.6)	2.00 (0.89 ‐ 4.62)	0.09
				
**Marijuana use in 30 days**				
0 times	22/131	(16.8)	1	
1 or more times	6/27	(22.2)	1.80 (0.70‐4.54)	0.22
				
	**Males reporting female partnerships**			
				
**Age (Years)**				
0‐19	4/11	(36.4)	1	
20‐29	12/25	(48.0)	1.62 (0.38 ‐ 6.94)	0.52
30 & Above	2/7	(28.6)	0.70 (0.09 ‐ 5.43)	0.73
				
**Ethnicity**				
White	0/4	(0.00)	1	
African American	11/42	(26.2)	0.4 (0.29‐31.23)	3.00
Hispanic or Latino	1/5	(20.0)	0.9 (0.03‐17.51)	0.75
Others	2/5	(40.0)	4.5 (0.25 ‐ 80.57)	0.31
				
**Marital status**				
Never been married	10/46	(21.7)	1	
Married	1/4	(25.0)	1.3 (0.17 ‐ 10.05)	0.80
Separated/Divorced/Widow	3/7	(48.9)	1.73 (0.35 ‐ 8.64)	0.502
				
**Age at sexual debut**				
< 19 years	10/48	(20.8)	1	
> 19 years	1/4	(25.0)	0.429 (0.04‐4.42)	0.48
				
**5 or more drinks of alcohol in last 30 days**				
0 days	7/36	(19.4)	1	
1 or more days	7/19	(36.8)	2.16 (0.66 ‐ 6.69)	0.18
				
**Marijuana use in last 30 days**				
0 times	8/37	(21.6)	1	
1 or more times	5/17	(29.4)	1.85 (0.58 ‐ 5.90)	0.30
				
**Use of illegal drugs and alcohol in last 30 days**				
0 times	10/47	(21.3)	1	
1 or more times	4/10	(40.0)	3.90 (0.71 ‐ 21.46)	0.12
				
	**Males reporting male partnerships**			
**Age (Years)**				
< 20	1/6	(16.7)	1	
20‐29	4/19	(21.1)	1.33 (0.12‐14.90)	0.82
30 & Above	0/3	(0.00)	0.00 (0.00‐0.00)	1.00
				
**Ethnicity**				
White	1/5	(20.0)	1	
African American	5/23	(21.7)	0.54 (0.70 ‐ 3.98)	0.53
Hispanic or Latino	1/4	(25.0)	0.50 (0.29‐8.95)2	2.64
Others	0/1	(0.00)	0.00 (0.00‐0.00)	1.00
				
**Marital status**				
Never been married	7/20	(23.3)	1	
Married	0/1	(0.00)	0.00 (0.00 ‐ 0.00)	1.00
Separated/Divorced/Widow	0/2	(0.00)	2.75 (0.15‐49.36)	0.49
				
**At least one drinks of alcohol in last 30 days**				
0 days	4/12	(33.3)	1	
1 or more days	2/9	(22.2)	3.50 (0.60 ‐ 20.41)	0.16
				
**Marijuana use in last 30 days**				
0 times	3/24	(12.5)	1	
1 or more times	3/7	(42.9)	17.50 (2.29‐134.28)	0.01
				
**Use of illegal drugs and alcohol in last 30 days**				
0 times	5/27	(18.5)	1	
1 or more times	2/6	(33.3)	8.80 (1.25 ‐ 62.19)	0.03
				
	**Females reporting female partnerships**			
				
**Age (Years)**				
0‐19	5/10	50.0	1	
20‐29	11/19	57.9	1.38 (0.30‐6.40)	0.69
30 & Above	3/4	75.5	3.00 (0.23‐39.61)	0.40
				
**Ethnicity**				
White	1/2	50.0	1	
African American	18/33	54.5	1.20 (0.07‐20.85)	0.90
Hispanic or Latino	1/1	100.0	-	1.00
Others	-	-	-	
				
**Marital status**				
Never been married	18/30	60.0	1	
Married	-	-	-	-
Separated/Divorced/Widow	2/5	40.0	0.44 (0.06‐3.07)	0.41
				
**At least one drinks of alcohol in last 30 days**				
0 days	8/16	50.0	1	
1 or more days	12/20	60.0	1.50 (0.40‐5.65)	0.55
				
**5 or more drinks of alcohol in last 30 days**				
0 days	13/24	54.2	1	0.23
1 or more days	7/9	77.8	2.96 (0.51‐17.29)	
				
**Marijuana use in 30 days**				
0 times	9/18	50.0	1	
1 or more times	8/14	57.1	1.33 (0.33‐5.43)	0.69
				
**Cocaine use in 30 days**				
0 times	19/33	57.6	1	
1 or more times	1/3	33.3	0.37 (0.03‐4.48)	0.43
				
**Use of illegal drugs and alcohol in last 30 days**				
0 times	14/26	53.8	1	
1 or more times	6/10	60.0	1.29 (0.29‐5.66)	0.74

* n is the number of respondents reporting multiple partnerships

N is the number of respondents with the listed characteristic. N varies based on the number of responses.

Among males who reported female partnership, those age 20‐29 years had the highest proportion (48%) reporting having two or more female partners in the previous 3 months. Among females who reported male partnerships, there were significant differences (p = 0.02) in the proportion reporting having two or more partners in the previous 3 months with the lowest (3.6%) and highest (31.3%) proportions being reported by respondent 30 years and older, and those below the age of 20 years respectively (Table 2). Tables also showed that female 30 years and older were significantly less likely (OR = 0.09, p = 0.03) to report having multiple male partners in the previous 3 months. For females who reported female partnerships, the lowest proportion reporting 2 or more partners in the last 30 days was the 30 years and older age group (Table 2). Overall, 40.1% of all the sexually experienced respondents reported multiple partnerships in the previous 3 months.

### Condom use

Table 4 shows the relationships between dependent variables (inconsistent condom use in the previous 30 days and not using condom during last sex) and selected variables. For this analysis, we separated the results by gender since the literature suggests differences in engagement in HIV risk behaviors between males and females. Using a condom always is coded as "consistent condom use" and other levels of condom use (rarely, sometimes, most of the time) or non-use as "inconsistent condom use."

**Table 4:** Factors associated with unprotected sexual intercourse among sexually experienced respondents by gender

Inconsistent condom use in previous 30 Days*					
	**Male**		**Female**		
	(%)^a^	Unadjusted Odds Ratio (95% CI)	(%)^a^	Unadjusted Odds Ratio (95% CI)	Adjusted Odds Ratio (CI)

**Age (Years)**					
0‐19	52.9	1	67.2	1	
20‐29	72.5	2.34 (0.72‐7.61)	83.1	2.41 (1.11‐5.20)**	
30 & Above	81.8	4.0 (0.66‐24.30)	88.5	3.74 (1.00‐13.95)**	
**Ethnicity**					
White	90.0	1	50.0	1	
African American	62.7	0.19 (0.02‐1.58)	77.4	3.42 (0.81‐14.35)	
Hispanic or Latino	42.9	0.08 (0.01‐1.07)	90.9	10.00 (0.84‐119.32)	
Others	80.0	0.44 (0.02‐9.03)	100.0	-	
**Marital status**					
Never been married	60.3	1	77.7	1	
Married	80.0	2.63 (0.28‐24.86)	91.7	3.16 (0.39‐25.29)	
Separated/divorced/widow	100.0		70.6	0.69 (0.23‐2.09)	
**Membership of fraternity**					
Yes	61.5	1	63.6	1	
No	66.2	1.22 (0.36‐4.16)	78.9	2.13 (0.59‐7.67)	
**Age of first sex initiation**					
< 19 years	58.8	1	78.8	1	
> 19 years	100.0	-	68.8	0.59 (0.19‐1.83)	
**At least one drinks of alcohol in last 30 days**					
No	60.6	1	71.8	1	
Yes	69.4	1.47 (0.58‐3.72)	81.6	1.74 (0.86‐3.50)	
**Marijuana use in last 30 days**					
No	61.4	1	74.3	1	1
Yes	80.0	2.51 (0.74‐8.50)	92.1	4.04 (1.17‐13.93)**	5.05 (1.34‐19.02)**
**Use if other illegal drug in last 30 days**					
No	66.3	1	78.1	1	
Yes	50.0	0.51 (0.03‐8.46)	66.7	0.56 (0.05‐6.33)	
**Alcohol and drug use in last 30 days**					
No	63.4	1	77.0	1	
Yes	81.8	2.60 (0.52‐12.96)	84.0	1.57 (0.51‐4.85)	
**Alcohol and/or drug use before or during last sex**					
Yes	81.8	1	71.4	1	
No	64.7	0.41 (0.08‐2.04)	78.0	1.42 (0.57‐3.51)	
Did not use condom last sex*					
	**Male**		**Female**		
	(%)^b^	Unadjusted Odds Ratio (95% CI)	(%)^b^	Unadjusted Odds Ratio (95% CI)	Adjusted Odds Ratio (CI)

**Age (Years)**					
0‐19	36.4	1	52.0	1	1
20‐29	52.9	1.97 (0.70‐5.50)	64.6	1.69 (0.93‐3.05)	1.61 (0.83‐3.13)
30 & Above	63.6	3.06 (0.86‐13.79)	78.8	3.43 (1.33‐8.86)**	3.86 (1.20‐12.47)**
**Ethnicity**					
White	53.3	1	45.5	1	
African American	45.9	0.61 (0.18‐2.09)	61.7	1.93 (0.57‐6.55)	
Hispanic or Latino	30.0	0.31 (0.52‐1.80)	84.6	6.60 (0.97‐44.93)	
Others	83.3	3.57 (0.31‐40.75)	70.0	2.80 (0.46‐16.93)	
**Marital status**					
Never been married	44.8	1	61.9	1	
Married	66.7	2.46 (0.43‐14.14)	83.3	3.08 (0.86‐11.01)	
Separated/divorced/widow	70.0	2.87 (0.70‐11.85)	52.2	0.67 (0.28‐1.60)	
**Membership of fraternity**					
Yes	52.6	1	41.7	1	
No	47.6	0.82 (0.30‐2.22)	63.7	2.45 (0.75‐7.98)	
**Age of first sex initiation**					
< 19 years	45.9	1	62.4	1	
> 19 years	63.6	2.06 (0.56‐7.58)	68.0	1.28 (0.53‐3.11)	
**At least one drinks of alcohol in last 30 days**					
No	45.5	1	54.2	1	
Yes	50.8	1.24 (0.57‐2.72)**	68.1	1.81 (1.06‐3.10)**	
**Marijuana use in last 30 days**					
No	46.7	1	61.7	1	
Yes	52.2	1.25 (0.49‐3.18)	65.9	1.20 (0.60‐2.39)	
**Use if other illegal drug in last 30 days**					
No	49.5	1	62.9	1	
Yes	25.0	0.34 (0.03‐3.38)	33.3	0.30 (0.03‐3.30)	
**Alcohol and drug use in last 30 days**					
No	47.8	1	61.8	1	
Yes	53.8	1.28 (0.40‐4.09)	67.9	1.30 (0.56‐3.02)	
**Alcohol and/or drug use before or during last sex**					
Yes	61.5	1	58.8	1	
No	48.8	0.60 (0.18‐1.97)	63.6	1.22 (0.58‐2.57)	

* Adjusted for all variables listed in the table.

^a ^Percent of sexually experienced students in each category who reported that they did not use condom always (inconsistent condom use) for sex in the previous 30 days.

^b ^Percent of sexually experienced students in each category who reported not using condom during last sexual intercourse.

** p ≤ 0.05

There were significant differences in condom use last sex (p = 0.01) and consistent condom use in the previous 30 days (p = 0.002) among the different age groups (Table 2). Seventy five percent of respondents 30 years and older, 61% of those age 20‐29, and 48.5% of those below the age of 20 years reported not using a condom last sex. The pattern was the same for always using a condom with 13.5% of those 30 years and older, 20.2% of 20‐29 year olds, and 35.9% of those below the age of 20 years reporting that they used condoms always in the previous 30 days.

When multivariate analysis were stratified by gender, there were no independent variables that were significantly correlated with condom use last sex and consistent condom use in the last 30 days among sexually active male respondents. Among females, those age 30 years and older were almost four times (OR = 3.74, p = 0.05) and those age 20‐29 years more than twice (OR = 2.41, p = 0.03) more likely to report inconsistent condom use in the previous 30 days compared to those below the age of 20 years. Also among sexually active females, those who reported using marijuana in the previous 30 days were 5 times more likely (OR = 5.05, p = 0.02) to report inconsistent condom use in the previous 30 days compared to those who did not (Table 4).

Age 30 years and older and reporting having at least at least one drink of alcohol in the previous 30 days significantly predicted not using a condom last sex among sexually active females. Those age 30 years and older were almost 4 times (OR = 3.43, p = 0.01) more likely to report that they did not use a condom last sex compared to those below the age of 20 years. Likewise, those who reported having a least one drink of alcohol in the previous 30 days were almost twice (OR = 1.81, p = 0.04) as likely to report not using a condom last sex compared to those who did not. When both male and female respondents were combined, females were almost two times more likely than males to report that neither they nor their partners used a condom the last time they had sex.

### Alcohol and drug use in the context of sexual intercourse

Alcohol and drug use alter judgment, remove inhibitions and engender high risk sexual behaviors. No significant differences were observed in alcohol and/or drug use in the context of sexual activity. The highest (14.6%) proportion reporting alcohol and/or drug use before their last sex was among respondents below the age of 20 years and the lowest (4.9%) among those 30 years and older (Table 2).

### Perception of HIV risk

The perception of the risk of HIV infection was generally low among the students in our sample. Fifty four percent of those age 30 years and older, 48.1% of 20‐29 year olds, and 57.9% of those below the age of 20 years perceived themselves as not having any chance of being infected with the HIV (Table 2). Only 46% of respondents reporting inconsistent use of condoms in the previous 30 days perceived themselves to have a moderate to good chance of being infected with the HIV virus. Proportion was 53.5% for those who reported multiple partnerships in the previous 3 months. However, respondents who reported marijuana, alcohol or other drug use in previous 30 days, used condom inconsistently, or had multiple partners were significantly more likely to report perceiving themselves to have a moderate to good chance of being infected with HIV (Table 5).

**Table 5:** Factors associated with reporting moderate to good perception of HIV risk

Variables	(%)^c^	Unadjusted Odds Ratio (95% CI)	Adjusted Odds Ratio (95% CI)
**Marijuana use in last 30 days**			
No	30.8		
Yes	51.4	2.38 (1.40‐4.04)**	
**Alcohol and/or drug use in last 30 days**			
No	31.7		
Yes	65.9	4.16 (2.10‐8.24)**	4.72 (1.80‐12.40)**
**Condom use last 30 days**			
Consistent	27.9		
Inconsistent	46.0	2.19 (1.21‐3.99)**	1.96 (1.01‐3.84)*
**Multiple partnership**			
No	37.0		
Yes	53.5	1.96 (1.17‐3.28)**	1.81 (1.01‐3.27)*

^c ^Percent of sexually experienced students in each category who reported that they perceived themselves to be at moderate/good risk for HIV infection.

* p ≤ 0.05; ** p ≤ 0.01

## Discussion

In this study, we examined the HIV sexual risk behaviors and HIV risk perception of college students in a predominantly African American, commuter urban university located in the Midwest. The findings confirmed the prevalence of high risk sexual behaviors and low perception of risk in this cohort. Significant differences in high risk sexual behaviors found in this study support the earlier recommendation by Lewis et al. [20] of the need to conduct cross-sectional studies among diverse student populations in different settings.

Having multiple sexual partners is a recognized HIV risk [37,38]. Therefore, reducing the number of sexual partners is a worthwhile HIV prevention strategy. Among never married respondents in our study, multiple partnerships and lack of condom use were very high. While multiple partnerships were prevalent among all "relationship types" examined, the prevalence was highest in male/female relationships with almost half of males reporting having two or more female sexual partners in the last 3 months. The rates of multiple partnership found in this study are lower than reported by Bazargan et al.[18]. This difference should be interpreted with caution as the Bazargan et al. study was conducted with a racially similar but younger population and other factors including residency and campus type may play some role. The findings of respondents 30 years and older of both genders being least likely to report multiple partnerships is consistent with report from a study by Opt and colleagues [39], which compared "traditional" (younger) and "non-traditional" (older) students in a predominantly White institution and found a lower percentage of non-traditional students reporting multiple partnerships. These findings may be attributed to the "maturing out" phenomenon. Overall, respondents who reported alcohol or illegal drug use in the last 30 days were more likely in all cases to have two or more partners. This once again suggests that individuals with one HIV risky behavior are more likely to exhibit others.

Condom use was largely low among sexually experienced respondents of all ages in our sample in our sample. The rates of consistent condom use in the last 30 days during sex are much lower than the 37% reported in studies [31-43] with predominantly White college students. The finding that respondents age 30 years or older were significantly more likely than those below 30 years to report that they or their partners did not use a condom during last sex and are less likely to report that they or their partners always used a condom during sex in the last 30 days was unexpected. The same age group was less likely to report having more than one sexual partner. As a result of this paradox, any protective effect conferred by having single partnership is eroded by lack of condom use because their partners may also have other partners. Females were also significantly more likely than males to report that they or their partners did not use a condom during last sex and less likely to report that they or their partners always used a condom during sex in the last 30 days. Low condom use among females is quite troubling because women are more likely than men to be infected with HIV during sexual intercourse. While we did not explore who the sexual partners of the respondents in our study are, we assume they will be mostly other students. Therefore, the tandem of high rates of multiple partnerships among males and low condom use among females are of public health concern.

Alcohol and drug use are believed to increase sexual risk taking. Several studies [8,44] including the National Youth Risk Behavior Survey [45] have documented this relationship. The use of marijuana, which is more prevalent among our sample than in national surveys of college students [46,47], was significantly correlated with inconsistent condom use, while having at least one drink of alcohol in the previous 30 days was correlated with not using a condom last sex among females. As a group, individuals who use illegal drugs, including marijuana, were also more likely to report having multiple sexual partners and inconsistent condom use. This observation is not surprising as previous studies [48,49] have shown that individuals engaging in HIV risk behaviors often engage in other high risk or illegal behaviors. These findings will indicate that more attention needs to be focused on the role of alcohol and drug use in the participation HIV sexual risk behaviors in the design and implementation of HIV prevention programs for college students.

Perception of risk may be a strong motivating factor for behavioral change, particularly if the individual perceives control over the risk behavior [50]. The tendency to systematically underestimate personal risk termed 'optimistic bias [51] and treating HIV infection as a distant possibility [52] have been reported among college students. Despite the high level high risk HIV sexual risk behaviors among our sample, self-perception of HIV was low. The risk here is that students might not take any HIV preventive measures as have a poor perception of their risk status. The only silver lining here was that students who reported marijuana, alcohol, or other drug use, inconsistent condom use, and multiple sexual partners were significantly more likely than those who did not to perceive themselves as having a moderate to good risk of being infected with HIV.

In an age in which the use of technology has improved communication and information delivery, college student television broadcasts, student cell phone number registration to transmit prevention messages periodically, use of blogs, students newspapers are all approaches that can be considered in designing HIV prevention interventions for college students. All these communication channels should emphasize the importance of condom use and minimizing the number of sexual partners. The use of computer based programs has been successfully implemented on some college campuses. In addition, providing free condoms in college or student residence hall bathrooms can be done. All these should occur only after an assessment of HIV prevention needs assessment on college campuses.

### Study limitations

This study has several limitations. First, because of the sensitive nature of the survey, findings are subject to social desirability bias. Second, there is the possibility of recall bias since respondents were expected to provide information on previous behaviors. Though, classes where the participating students came from were randomly selected, not all the instructors responded to our request to survey the students, and 7% of students in the classes where surveys were done refused participation. The students who did not participate might be different in their sexual behaviors from those who participated. Finally, because of the sample size and the fact that selected courses were general education courses, generalization of findings should be done with caution. A study with a larger sample involving all students will provide more information.

In spite of the stated limitations, the findings from our study have several implications for the design and implementation of HIV prevention interventions on college campuses. It is obvious that a "one size fits all" approach cannot be taken. The low rates of condom use among females may be due to poor condom negotiating skills, poor condom use self-efficacy or apathy to the HIV/AIDS epidemic, a combination of several factors and needs further investigation. Alcohol and drugs were not a major factor in the participation of males in HIV sexual risk behaviors but marijuana use was correlated with inconsistent condom use and alcohol use with not using condom last sex for women. It is therefore important that HIV prevention interventions for female college students incorporate drug and alcohol education, particularly in the context of sexual activity.

### Future directions

The age, gender, and marital status differences observed in our study need further study. The role of communication regarding sex and HIV prevention including HIV testing, skill-building, condom use efficacy, and refusal self-efficacy related to incidental sex and alcohol/drug use in the context of sex need further study. Interventions designed specifically for older students that focus on condom promotion and education need to be implemented and evaluated. Condom education that focuses on condom negotiation skills and self efficacy should be implemented and evaluated for African American college students. In addition, given the importance of our findings on correlation of alcohol and drugs with unsafe sexual practices, further research to explore the context of substance use behaviors is recommended.

## Conclusion

The students in our study had several risk factors for HIV infection and transmission and paradoxically poor risk perception for HIV infection. Their engagement in HIV sexual risk behaviors varied by age and gender. Condom use was generally poor for older students, while alcohol and drug use was important for women. The findings suggest that college students can not be considered a homogenous population for which one type of intervention will be effective. This is particularly true for colleges with older non-traditional students. These older non-traditional students, particularly those who have full-time employment like those in our study sample, might not be receiving HIV prevention interventions in their communities or place of work. The college environment therefore provides opportunities to reach them with HIV prevention interventions.

## Competing interests

The authors declare that they have no competing interests.

## Authors' contributions

ASA participated in the design of the study and wrote the manuscript. TCA supervised the implementation of the study. Both ASA and TCA performed the analysis of the data. JAB revised the manuscript for content. MLD participated in the design of the study and obtained necessary institutional review approval for the study.

## Pre-publication history

The pre-publication history for this paper can be accessed here:

http://www.biomedcentral.com/1471-2458/9/281/prepub
